# Influence of the incorporation of triclosan methacrylate on the physical properties and antibacterial activity of resin composite

**DOI:** 10.1590/1678-7757-2018-0262

**Published:** 2019-08-30

**Authors:** Andreia Bolzan de Paula, Roberta Caroline Bruschi Alonso, Jesus Roberto Taparelli, Jéssica Rodrigues Camassari, Lúcia Helena Innocentini-Mei, Lourenço Correr-Sobrinho, Regina M. Puppin-Rontani

**Affiliations:** 1 Universidade Estadual de Campinas Universidade Estadual de Campinas Faculdade de Odontologia de Piracicaba Departamento de Odontologia Restauradora São Paulo Brasil Universidade Estadual de Campinas, Faculdade de Odontologia de Piracicaba, Departamento de Odontologia Restauradora, Área de Materiais Dentários, Piracicaba, São Paulo, Brasil.; 2 Universidade de Mogi das Cruzes Universidade de Mogi das Cruzes Faculdade de Odontologia São Paulo São Paulo Brasil Universidade de Mogi das Cruzes, Faculdade de Odontologia, São Paulo, São Paulo, Brasil.; 3 Universidade Estadual de Campinas Universidade Estadual de Campinas Faculdade de Engenharia Química Departamento de Materiais e Bioprocessos Campinas São Paulo Brasil Universidade Estadual de Campinas, Faculdade de Engenharia Química, Departamento de Materiais e Bioprocessos, Área de Ciência e Tecnologia de Materiais, Campinas, São Paulo, Brasil.; 4 Universidade Estadual de Campinas Universidade Estadual de Campinas Faculdade de Odontologia de Piracicaba Departamento de Odontologia Infantil Piracicaba São Paulo Brasil Universidade Estadual de Campinas, Faculdade de Odontologia de Piracicaba, Departamento de Odontologia Infantil, Área de Odontopediatria, Piracicaba, São Paulo, Brasil.

**Keywords:** Triclosan, Composite resins, Surface properties, Hardness, Anti-bacterial agents

## Abstract

The incorporation of antimicrobials in the composites as an attempt to reduce bacterial adhesion without jeopardizing mechanical properties is a challenge for Dentistry.

**Objective:**

To evaluate the bacterial adhesion and physical properties of a composite containing the methacrylate triclosan- derivative monomer (TM).

**Methodology:**

TM was synthesized and added to an experimental composite. Samples were divided into two groups: Control and TM (13.4 wt%). Antibacterial Activity: Three specimens of each material were prepared and placed on bacterial suspensions of *Streptococcus mutans* for 1, 5 and 10 days. After these periods the counting of the colonies (log10) was performed. Assays was performed in triplicate. Physical Properties: Three-body Abrasion (TBA): Ten specimens of each material were prepared and stored at 37°C/24 h. The surface roughness (Ra) and hardness (KHN) were analyzed. Next, the specimens were submitted to abrasive wear (30,000 cycles) and re-evaluated for Ra and KHN; Sorption/solubility (SS): cylindrical specimens (n=10) were prepared and weighted. The specimens were immersed in deionized water for 7 days at 37°C and then their weight was verified again. SS were calculated using accepted formulas; Diametral tensile strength (DTS): specimens (n=10) underwent test performed in an Instron universal testing machine at a crosshead speed of 1 mm/min. Data were submitted to appropriate statistical tests according to data distribution and assay (p<0.05).

**Results:**

Bacterial Adhesion: TM showed a significant reduction on biofilm accumulation in the evaluated periods: 1 day (1.537±0.146); 5 days (2.183±0.138) and 10 days (4.469±0.155) when compared with Control: 1 day (4.954±0.249); 5 days (5.498±0.257) and 10 days (6.306±0.287). Physical Properties: For TBA, SS and DTS no significant difference was found between groups Control and TM. The incorporation of methacrylate triclosan-based monomer in the experimental composite reduce bacterial adhesion of *S. mutans* and did not affect important polymer properties.

## Introduction

Secondary caries are the most common reason for replacing dental restorations, presumably related to biofilm formation on and within gaps at the restoration margins,^1^ in this way, antibacterial properties of restorative materials are of major clinical importance and would allow dental tissue preparation, positively influencing the treatment outcome, especially for high caries-risk patients.^2^ To reduce the proliferation of microorganisms on the tooth-composite interface and around dental restorations, various chemical compounds have been added to the materials composition.^3^^,^^4^

Restorative resin-composites, due to surface roughness and residual monomers release after polymerization,^5^ favor bacterial colonization much more than other dental materials, such as amalgam, ceramics, gold alloys, or glass ionomer cements.^6^ Additionally, bacteria present in the biofilm also induces further microorganism adhesion to composite filling,^7^ leading to surface softening and increase roughness of the composite.

Sing, et al.^8^ (2013) and das Neves, et al.^9^ (2014) evaluated the incorporation of antibacterial agents, such as chlorhexidine and silver nanoparticles in the organic matrix of resin-composites. For these compounds, antibacterial activity was effective, however it occurred though the release of antibacterial agents.^8^^,^^9^ The release of this agents can be toxic or harm the mechanical properties of the restorative material.^10^ Triclosan (2,4,4’-trichloro-2’-hydroxydiphenyl ether) is an antibiotic widely used in oral hygiene products, such as mouthwash and toothpaste, due to its safe profile and broad spectrum of activity.^11^ Toxicologycal studies showed that triclosan and its metabolites are well tolerated by human beings.^12^^,^^13^ De Salva, et al.^13^ (1989) showed that triclosan is not a mutagen, carcinogen, or teratogen agent and is safe in reproductive studies.^13^

Triclosan has been incorporated into restorative material in powder form, although antibacterial activity was observed, the incorporation caused further degradation of the restorative material because it was leached.^14^^,^^15^ In this way, the incorporation of a triclosan-based monomer into the resin matrix of composites would be more appropriate, since it can reduce bacterial adhesion by contact on the restoration surface without reducing mechanical properties over time, which can improve the long-term performance of the restorative material.

Also, we should consider that, in addition to the chemical challenges due to acids from the biofilm fermentation process, restorative materials are also constantly subject to mechanical challenges in the oral cavity.^16^ Daily toothbrushing of the already damaged surface causes gradual loss of the softened material (matrix and filler), leading to color change, contour loss, and increase roughness of the restoration surface again, influencing its aesthetic and clinical longevity.^17^

For resin-composites, hydrolysis can negatively affect physical and mechanical properties, what is particularly important for restorative dental composites, since they are always exposed to the water present in the oral environment.^18^^,^^19^ The chemical resistance of these materials is related to the quality of polymerization and to the chemical characteristics of the monomers present in the resin matrix.^20^ In this way, the evaluation of sorption and solubility is very important, since these phenomenon are related to hydrolytic degradation of resin materials and may serve as precursors to a variety of chemical and physical processes, such as swelling, plasticization, oxidation and hydrolysis.^19^^,^^20^

In this context, the incorporation of a triclosanbased monomer into the resin matrix for dental composites would be very interesting, as long as it reduced biofilm formation without altering the properties of the material, which can improve the long-term performance of the restorative material in the oral cavity. Thus, the aim of this study is to evaluate the bacterial adhesion and physical properties of a composite containing the monomer based on triclosan methacrylate.

## Methodology

### Formulation of the composites

Triclosan-methacrylate (TM), was obtained by the esterification of triclosan, i.e. 2,4,4-trichloro hydroxy diphenyl ether supplied by Sigma Aldrich, St. Louis, MO, USA, with methacrylic acid in dimethylformamide (DMF) solution. In this study, two low viscosity composites were formulated:(C) Control composite and; (TM) Composite containing TM monomer. For both composites, a resin matrix with Bisphenol A ethoxylate dimethacrylate (BisEMA – Sigma-Aldrich, Inc., St. Louis, MO, USA, Batch #03C514HF), and triethylene glycol dimethacrylate (TEGDMA – Sigma-Aldrich Inc., St. Louis, MO, USA, Batch #01612M), were prepared in the proportion 6:4.^21^ For TMC, the TM monomer was incorporated into this resin matrix at 14.4%wt (percentage determined in a pilot study). After, for both materials, the photoinitiator BAPO [Phenylbis (2,4,6-trimethylbenzoyl) phosphine 511447-10g Lot #MKBJ9151V] by Aldrich Chemistry, St. Louis, MO, USA, was incorporated at 2wt% and the inhibitor dibutylhydroxytoluene (BHT) was incorporated at 0.1wt%. Both resin matrix were reinforced with silanized barium aluminum silicateglass fillers in two different sizes: (1) BaAlSi ultrafine – 1 µm (GM27884 Lot #Sil4387, SCHOTT) at 40 wt% and (2) BaAlSi nanofine – 180 nm (GM27884 Lot #Sil4232, SCHOTT) at 10%, both from Electronic Packaging GmbH, Landshut, Germany. The fillers were added incrementally and mixed homogeneously to a 50 wt% loading using a high-speed mixing machine (SpeedMixer™ DAC 150.1 FV, Hauschild, SC, USA). The experimental composites were manipulated under filtered orange light. The composition of the experimental composites formulated for this study were based on a previous study.^22^

For all tests, the experimental composites were photoactivated for 40 s, using a Bluephase LED light curing unit (Ivoclar Vivadent, Schaan, Liechtenstein) with a power density of 1,200 mW/cm^2^. A radiometer (Hilux Dental Curing Light Meter, Benlioglu Dental Inc., Demetron, Ankara, Turkey) was used to verify the power density of the curing unit.

### Bacterial adhesion assay

Strains of *Streptococcus mutans* UA159 were used in this study. To prepare the inoculum, *S. mutans* was first grown on Mitis Salivarius agar (Difco Laboratories, Sparks MD, MI, USA) plates at 37°C for 48 hours supplemented with 10% CO_2_. Subsequently, single colonies were inoculated into 5 mL of brain heart infusion (BHI) broth (Difco Laboratories) and incubated at 37°C for 18 hours. The culture was adjusted to an optical density of 0.6 at 550 nm (approximately 3x10^11^ CFU/mL) and diluted 1:20 in BHI supplemented with 0.1% sucrose. Circular specimens (5 mm diameter × 1 mm thick) of each material (C and TM) were prepared (n=3) in a polyvinyl siloxane mold (Aquasil, Dentsply DeTrey, Konstanz, Germany) covered with a mylar strip. Specimens were not submitted to polishing, presenting smooth surface without bubbles or defects. All the specimens were sterilized with ultraviolet light for 20 minutes. The specimens were kept in static culture medium containing 1% sucrose for 1, 5 and 10 days (37°C/10% CO_2_). The medium was renewed every 24-hours. Next, the specimens were washed for 5 minutes in RTF solution to remove the non-adhered bacteria. The bacterial suspensions obtained were serially diluted in MSA.^21^ The purity of the cultures in the media was verified every day using Gram staining and plating samples. For the adhesion assay three independent experiments were performed in triplicate. The data were transformed into Log_10_ and submitted to two-way ANOVA (α=0.01), considering material (2 levels: C and TM) and time (3 levels: 1 day, 5 days and 10 days), using Bioestat 5.0. Means were compared by the Tukey's test.

### Simulated toothbrusing

Ten specimens of each composite (5-mm diameter × 2-mm thick) were prepared in plastic molds, covered with a transparent polyester strip and photocured for 40 s. All specimens were maintained in the dark at 100% relative humidity and at 37°C for 24 hours. The surfaces were wet polished with silicon carbide abrasive papers 600-, 1200-, and 2000-grit (Norton SA, São Paulo, SP, Brazil) in an automatic polisher (APL4; Arotec, Cotia, SP, Brazil) to obtain a flat surface. After, specimens were ultrasonically cleaned (Ultrasonic Cleaner, model USC1400, Unique Co, São Paulo, Brazil) in distilled water for 10 minutes to remove polishing debris.

All specimens were analyzed for surface roughness and Knoop hardness (baseline measures). For surface roughness testing, the specimens were analyzed using a Surfcorder SE1700 instrument (Kosaka Corp, Tokyo, Japan), with cutoff length of 0.25 mm, at a tracing speed of 0.1 mm/s. The mean surface roughness values (R_a_), mm of each specimen were obtained from three successive measurements of the center of each disk in different directions, and the Ra mean was calculated for each disk. Next, Knoop hardness measurements were performed using a microhardness tester (HMV-2; Shimadzu Corp., Tokyo, Japan) under a load of 50 g for 15 s. Three indentations were made for each specimen, and the average value of the three readings was considered.

In order to suppress abrasive degradation of the surfaces, brushing simulation was conducted at 250 cycles/minute, for 30,000 cycles with a 200-g load on all the groups. Colgate Total dentifrice (Colgate Palmolive Ind e Com Ltda, São Bernardo do Campo, SP, Brazil) diluted in distilled water (1:2) was used as abrasive third body. Next, specimens were washed in an ultrasonic bath for 10 minutes, gently dried and reevaluated for roughness and hardness. Data were submitted to two-way repeated ANOVA measures, considering Material (2 levels: C and TMC) and evaluation period (2 levels: before abrasion and after abrasion), and means were compared using Tukey's tests with a 5% significance level.

### Sorption and solubility test

Sorption and solubility evaluation were performed according to Inagaki, et al.^23^ (2016). Specimens (7 mm diameter x 1 mm thickness) (n=5) of each material were prepared in a polyvinyl siloxane mold (Express XT, 3 M ESPE, St. Paul, MN, US). The diameter of 7 mm was selected based on the active tip of the light curing device, in order to promote homogeneous polymerization. To obtain a smooth and standard flat surface of the specimens, a polyester strip was placed over and covered with a glass slide until the light curing process. Each disk was light cured for 40 s. After light curing, the disks were individually dry stored in closed Eppendorfs at 37°C for 24 h. After this period, the Eppendorfs containing the disks were opened and placed in a desiccator containing silica gel in a vacuum environment at 37°C for 24 h. Then each disk was weighed in an analytical balance (Tel Marke, Bel Quimi, São Paulo, SP, Brazil) with an accuracy of 0.001 g. This cycle of drying in silica and weighing in a balance was repeated until the constant mass (M1) of each disk was obtained.

Immediately after establishing M1, the diameter and thickness of disks were measured in two different directions using a digital caliper (0.01–150 mm, Product Code 500-144B, Mitutoyo, Tokyo, Japan) with an accuracy of 0.01 mm. The mean value of diameter and thickness were obtained to calculate the volume of the cylinder (V), in cubic millimeters (mm^3^). Then the disks were immersed, individually, in falcon tubes with 4.66 mL of deionized water at 37°C for 7 day. After 7 days, the disks were removed from the water and dried in absorbent paper for 15 s. One minute after being removed from the water, each disk was weighed only once in an analytical balance to obtain the mass (M2). After this, the disks were placed again in a vacuum desiccator with silica gel, and the cycle aforementioned was performed until they kept a constant mass (M3). The values (in mg/mm^3^) of water sorption (Wsp) and solubility (Wsl) were calculated using the following equation: Wsp=(M2 - M3)/V and Wsl=(M1 - M3)/V. Data were subjected to the Mann-Whitney test (*p*<0.05), using the software ASSISTAT Version 7.7 en (2017) (http://www.assistat.com; UFCG, Campina Grande, Brazil).

### Diametral tensile strength (DTS)

For DTS testing, ten cylindrical specimens (4 mm diameter x 2 mm height) of each composite (C and TM) were prepared in a teflon mold. Specimens (n=10) were placed in the mold between two polyester films and cured from both sides for 40 s. Specimens were carefully removed by unscrewing the split mold and were subsequently finished to remove any irregularities using 1000-grit abrasive papers (Norton SA). After 24 h the storage at 37°C, DTS was measured using a universal testing machine (Instron, model 4411, Canton, MA, USA) at a crosshead speed of 1 mm/minute. The DTS value in MPa was calculated using the following Eq: DTS=2F/πdt, where F is the maximum force applied to failure (N), d is the diameter (4 mm) and t is the thickness (2 mm). Data were subjected to normality tests with the Shapiro Wilk test. After, all data were submitted to one-way ANOVA and t-test (α=0.05) using the software ASSISTAT Version 7.7 en (2017).

## Results

Table 1 shows the mean values and standard deviation of *S. mutans* adhesion to the surface of the experimental materials (C and TM) after 1, 5 and 10 days. The incorporation of the triclosan methacrylate monomer in the experimental composite (TM) significantly reduced the adhesion of *S. mutans* on the surface of the material in all periods when compared with the control composite (C), evidencing the antibacterial effect of TM monomer over time. However, after 10 days of biodegradation a significant increase was observed in the adhesion of *S mutans* on the surface of the TM when compared with days 1 and 5.

**Table 1:** Means ± standard deviation of bacterial adhesion of *Streptococcus mutans* (UFC/ml (log10) in surfaces of the composites Control and TM after 1, 5 and 10 days

Bacterial adhesion												
Materials		1 day				5 days				10 days		
Control	4.954	(0.249)	^a^	^a^	5.498	(0.257)	^a^	^A^	6.306	(0.287)	^a^	^A^
TM	1.537	(0.146)	^a^	^B^	2.183	(0.138)	^a^	^B^	4.469	(0.155)	^b^	^B^

Means followed by different uppercase letters in the same column and lowercase in the same row indicate significant differences for composites (p<0.05)

Table 2 shows the mean Ra and KHN values and the standard deviations, before and after abrasion. No significant difference was found between C and TM for Ra and KHN values, before or after abrasion; however, for both materials a significant increase was observed in Ra and KHN values after abrasion.

**Table 2:** Surface Roughness means (µm) and Knoop hardness (KHN) and standard deviation standard of the composites Control and TM submitted to abrasion

Materials	Roughness						Knoop Hardness					
	Initial			After abrasion			Initial			After abrasion		
Control	0.1865	(0.07)	^aA^	0.3798	(0.9)	^aB^	44.2	(4.2)	^aB^	55.0	(7.0)	^aA^
TM	0.1924	(0.05)	^aA^	0.4051	(0.11)	^aB^	39.5	(4.8)	^aB^	56.0	(8.7)	^aA^

Means followed by different lowercase letters in the same column and uppercase in the same row indicate significant differences for composites (p<0.05)

Table 3 shows median, minimum and maximum values of sorption/solubility (mg/mm^3^) and diametral tensile strength (MPa) ± standard deviation of composites Control and TM. For both tests, no significant difference was found between C and TM.

**Table 3:** Median, minimum and maximum values of sorption/solubility (mg/mm^3^) and Diametral Tensile Strength (MPa) of composites Control and TM

Materials		Sorption			Solubility		Diametral Tensile Strength
	**Median**	**Mínimum**	**Máximum**	**Median**	**Mínimum**	**Máximum**	
Control	0.02212	0.00398	0.02981^a^	0.00000	-0.02685	0.01437^a^	586.20 ± 97.78^a^
TM	0.02710	0.00413	0.04206^a^	0.00000	-0.00817	0.00826^a^	620.42 ± 99.01^a^

Means followed by same lowercase letters in the same column indicate no significant differences for composites (p>0.05)

## Discussion

Biofilm growth around restorations causes pH decrease and consequently a recurrent process of demineralization.^4^ In order to reduce restoration failing, antibacterial agents have been added to dental materials.^7^^–^^9^ Triclosan has been incorporated into different polymers with wide degrees of success.^24^ In this study, it was observed that the composite containing triclosan-methacrylate had inhibitory effects on bacterial adhesion in all evaluated times.

Triclosan was thought to reduce bacterial growth and polymer adherence directly from the polymer surface.^24^ This bacterial adhesion inhibition can be explained by the triclosan molecular mechanical agitation on weak bacterial membranes. Further, bacterial inhibition includes possible membrane structural bond rotation entanglements with secondary bonding defects. Both forms of bacterial inhibition can be disruptive, particularly during the growth log phase, when actively dividing cells require correct membrane fluidity.^15^ Also, secondary bonding between bacteria and polymer was interrupted by triclosan vibrational fluctuating mechanomolecular bond rotations as a possible mechanism to prevent microbial surface attachments.^15^^,^^24^

Despite the details of the mechanism of action of triclosan-methacrylate monomer remains undetermined, it is possible that it takes place by contact, considering that the element chlorine (Cl), a constituent of the triclosan molecule, is fixed in the polymer network after curing. Due to the presence of Cl, the immobilized agent may induce inactivation of glucosyl transferase in bacteria reducing plaque growth.^25^

Brecx, et al.^25^ (1983) demonstrated that plaque growth is the result of the proliferation of bacteria already present on the surface and of a continuous deposition of additional bacteria. Therefore, a lower rate of bacterial growth and a lower rate of glucan synthesis would be responsible for the significantly lower bacterial adhesion on the experimental composite^21^ compared with the control within 24 hours, 5 and 10 days. However, according to the results of this study, bacterial adhesion in the TM containing composite have increased after 10 days, what can demonstrate that this material presents limited antibacterial activity, which will be evaluated in future studies.

Even so, the use of methacrylate triclosan monomer immobilized/attached to the polymer network would be advantageous, since it provides the antibacterial effect of triclosan and may prevent voids within the polymer structure. When the antibacterial compound is not attached to the polymer, it is leached,^15^ facilitating the hydrolytic degradation of the material. In this matter, further studies must be carried out, focusing on the long-term biodegradation of the material.

A resin composite must show satisfactory mechanical properties to be accepted as a successful restorative dental material. Mechanical brushing is suitable for simulating normal oral hygiene procedures to standardize brushing application, distance and frequency of force on the specimen.^26^ There is no agreed clinical threshold for unacceptable values for the roughness of resin composites. However, Bollen, et al.^27^ (1997) reported that a material incapable of attaining and/or maintaining the Ra value below 0.2 µm would be susceptible to an increase in plaque accumulation, with higher risk of caries and periodontal inflammation^27^. Regarding the low viscosity composites tested in this study, there was no difference between the tested materials. both showed Ra values lower than 0.2 µm before abrasion and higher than 0.2 µm after abrasion.

Clinically, increasing the surface roughness of restorative materials influences the aesthetic characteristic of the restoration, due to discoloration and wear. Also, the periodontal health may be affected, since higher roughness favor biofilm accumulation, which may cause gingivitis. The development of secondary caries may also be a consequence of biofilm accumulation. In this study, brushing abrasion was conducted since it can contribute to the disintegration of dental materials. The surface of the compound disintegrates over time, which leads to roughness, staining and increased wear.^28^

In this study, both groups showed increased surface roughness after brushing. The brushing simulator was used to demonstrate wear behavior, resulting in wear of the organic matrix and of the filler particles, which were exposed and protruded,^29^ increasing the surface roughness values. In addition, the formulation of the composite also affects its surface roughness.^30^ The monomer TEGDMA is somewhat hydrophilic when compared to BisGMA and its presence in the formulation of resin composites has been associated with increased water sorption^31^ and consequently with the material degradation, which was accelerated by brushing. Still, similar values of roughness were observed for both groups before and after abrasion, showing that the incorporation of the TM did not affect the surface smoothness of the material against mechanical wear.

The hardness of resin composites depends on the filler type and content, and it has been suggested to correlate with other mechanical properties, such as abrasion resistance.^29^ In this study, similar hardness values were observed for both groups before and after abrasion. The same filler type and content (50 wt%) may have contributed to similar hardness values between control and TM groups. Before abrasion, both groups presented lower KHN than after. It is possible that the polyester strip and microscope slide load on the composite during specimen preparation lead inorganic fillers to concentrate at the center of the composite disc.^32^ Therefore, the resin monomer would emerge to the top region and would lead to lower KNH in the surface of the specimens. After abrasion, both groups showed increased hardness, due to the removal of this resin-rich superficial layer. Abrasion commonly takes place through a gradual removal of the softened organic material.^33^ It is suggested that this removal may lead to the exposure of particles, which may be responsible for the increase in the material hardness.

The properties of the resin composites are also influenced by the water present in the oral environment^34^ due to water sorption. Water causes hydrolysis, which may cause swelling and degradation of the organic matrix in resin composites.^35^ It occurs because hydrogen bonds are formed between water and polar groups present in polymers, such as hydroxyl and carbonyl, disrupting entanglements and secondary bonding between polymer chains.^23^ In this context, water acts like a solvent, leading to plasticization, changing the polymer molecular structure and increasing the mobility of polymer chain segments.^19^^,^^34^

Also, when polymers are immersed in water, some of the components, such as unreacted monomers, are dissolved and released.^33^ In this way, it was a concern that the incorporation of the TM monomer could increase the water sorption/solubility. According to Inagaki, et al.^23^ (2016), the chemical characteristics of monomers have more influence on the behavior of the materials than on the antimicrobial additive. Providentially, in this study, water sorption and solubility of the materials were not affected by TM presence

Regarding the diametral tensile strength, similar results were found for Control and TM groups, reinforcing that the incorporation of TM does not negatively affect the mechanical properties of the material and that the antibacterial monomer reacted properly with the other monomers of the composite. The immobilization of TM into the resin matrix promotes higher durability and increases the antibacterial capability of the material, without jeopardizing the mechanical properties.^15^ Other study^36^ evaluated the mechanical properties of a novel furanone-containing antibacterial resin composite and showed that the modified resin composite might be a clinically attractive dental restorative due to its high mechanical strength.

The composite containing the new antibacterial monomer showed satisfactory results since it reduced biofilm accumulation over days and maintained the physic-mechanical properties, suggesting the future possibility of using the TM antimicrobial monomer for clinical application.

## Conclusion

Within the limitation of this *in vitro* study, the results showed that the incorporation of triclosan-methacrylate to resin composites was able to reduce bacterial adhesion of *S. mutans* and decrease the formation of bacterial biofilm over 10 days. The incorporation of triclosan methacrylate did not significantly affect the surface hardness and roughness, before and after brushing, of the experimental composite. Sorption, solubility and diametral tensile strength of the material were maintained, being promising as a dental resin with intrinsic antibacterial action and suitable properties.
